# Supplementary material for: Identification of photoperiod-induced specific miRNAs in the adrenal glands of Sunite sheep (Ovis aries)
Source: Front Vet Sci. 2022 Jul 22;9:888207. doi: 10.3389/fvets.2022.888207 (PMC9354845; doi:10.3389/fvets.2022.888207)
Supplement: Supplementary file 1 [file Data_Sheet_1.ZIP › Supplementary file/miRDeep2 file.zip!/miRDeep2 file/Know_Structure/SP42A3/oar-mir-668.pdf]

miRBase precursor : oar-mir-668
Total read count : 18
oar-miR-668-5p read count : 0
oar-miR-668-3p read count : 18
remaining reads : 0

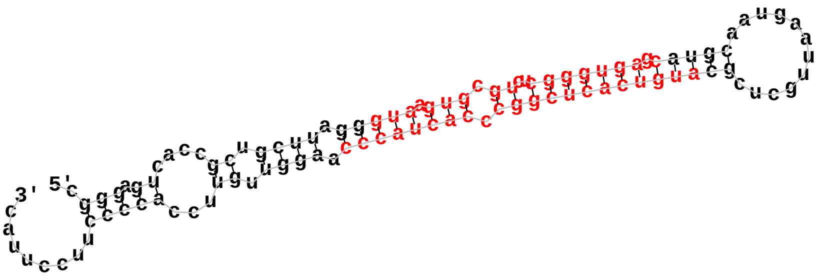

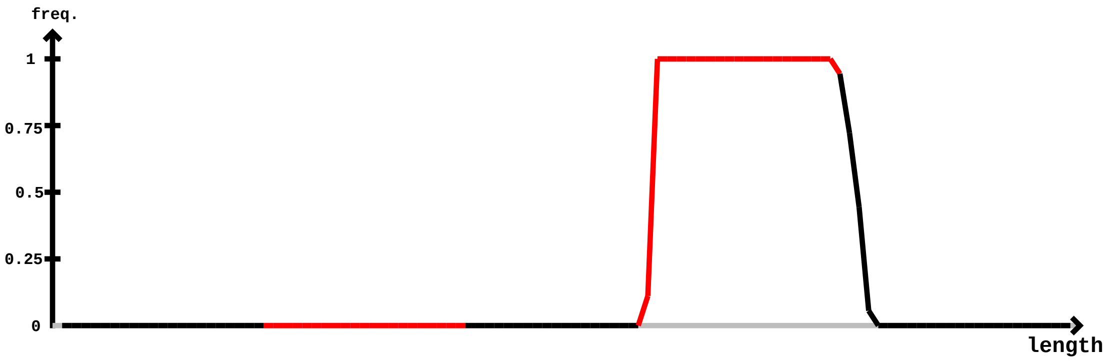


```
                                                                            oar-miR-668-3p
                                         oar-miR-668-5p
5'- cgggagucaccgcugcuuaggguaagugcgucucgggugagcaugcaaugaauugcucgcaugucacucggcccacuacccaagguuguuccaccccuuccuuac   -3'    exp
    .(((.((....((.((((.(((((.(((.((..(((((((.(((((............))))))))))))))).)))))))).)))).))...))))).........   reads    mm    sample
    ............................................................augucacucggcccacuaccca.......................   2        0     seq
    .............................................................ugucacucggcccacuacc.........................   1        0     seq
    .............................................................ugucacucggcccacuaccc........................   4        0     seq
    .............................................................ugucacucggcccacuaccca.......................   3        0     seq
    .............................................................ugucacucggcccacuacccaa......................   7        0     seq
    .............................................................ugucacucggcccacuacccaag.....................   1        0     seq
```
